# Cognitive Assessment for Neuropsychological Impairments in Dogs (CANID): An Efficient, Repeatable, and Sensitive Evaluation for Age-related Cognitive Dysfunction

**DOI:** 10.21203/rs.3.rs-9360671/v1

**Published:** 2026-04-27

**Authors:** Stephanie H. Hargrave, Emily E. Bray, Lorelei R. Switzer, Stephanie McGrath, Gene E. Alexander, Theadora Block, Elizabeth Carranza, Naomi Chao, Laura E. L. C. Douglas, Janet Galante, Emily Hilyard, Dara Jonkoski, Brenda S. Kennedy, Emma Kristal, Julie A. Moreno, Daniel E. L. Promislow, David A. Raichlen, Noah Stetson, Kaitlyn Tereso, Nova Vogel, The Dog Aging Project Consortium, Evan L. MacLean

**Affiliations:** University of Arizona; University of Arizona; University of Arizona; Colorado State University; University of Arizona; Canine Companions; University of Arizona; University of Arizona; Canine Companions; Sit! Stay! Play!; Sit! Stay! Play!; University of Arizona; Canine Companions; University of Arizona; Colorado State University; Tufts University; University of Southern California; University of Arizona; University of Arizona; University of Arizona; University of Arizona

## Abstract

We introduce the Cognitive Assessment for Neuropsychological Impairments in Dogs (CANID), a functional evaluation consisting of three cognitive tasks that measure aspects of memory and executive function. CANID can be implemented in a single ~ 40-minute evaluation, was designed for studies of cognitive aging in community dwelling dogs, and is sensitive to mid-life mild cognitive dysfunction. We developed a scoring system using a baseline sample (N = 239) and repeat testing at six (N = 140) and twelve (N = 118) month follow-up. Overall CANID scores had substantial repeatability at six (ICC = 0.63, 95% CI [0.51, 0.72]) and twelve (ICC = 0.59, 95% CI [0.47, 0.70]) months, as did all task-specific subscores. CANID scores at 6-month follow-up suggested modest practice effects (0.23 SD improvement in overall score relative to baseline), similar to those reported in human studies, but we did not find evidence for practice effects at one-year follow-up. To facilitate CANID’s use, we provide detailed protocols, video libraries, data recording instruments, experimenter training guidelines, and R code for scoring in an Open Science Framework repository.

## INTRODUCTION

Dogs are a valuable natural model for human cognitive decline and dementia given that many dogs spontaneously develop age-related cognitive impairment, which is sometimes accompanied by neuropathological changes resembling features of Alzheimer’s disease.

In studies with laboratory populations, cognitive impairments have been measured using standardized behavioral assays, which can detect mild cognitive impairment that often begins in midlife (Studzinski et al., 2006). However, these tests are labor intensive and rely on operant training protocols that are not feasible for large populations of community-dwelling companion dogs. Among companion dogs, cognitive aging and dementia has predominantly been studied using owner-report questionnaires (Madari et al., 2015; Salvin et al., 2011). Though valuable and highly scalable, these approaches do not provide direct functional assessments of cognition, and instead measure behavioral signs of dementia, which may only emerge in later stages of decline and when behaviors become apparent to owners. Thus, there is an important need for objective functional assessments of cognition that can detect early stages of cognitive dysfunction and which can be easily administered within large samples of companion dogs. Toward this aim, several sets of measures have been developed and used in recent studies of companion dog aging.

Previously, we developed the preliminary version of a test battery intended to capture age-related cognitive decline in companion dogs; for the present study, we aimed to refine this battery and evaluate its test-retest reliability. In our prior study, we reported results from a novel battery of five spontaneous problem-solving tasks measuring aspects of spatial learning and memory, response flexibility, and social behavior (Hargrave et al., 2025). In a diverse sample of companion dogs, age-related impairment was detectable in middle age (~ 8 years of age), and on average, older dogs performed substantially worse on measures of spatial memory, spatial reversal learning, and inhibitory control. We also demonstrated feasibility for these tasks in clinical settings. However, the 5-task battery was relatively lengthy, requiring administration across two 30–60-minute sessions. Additionally, our analyses were cross-sectional, precluding assessment of repeatability or practice effects, both important considerations for longitudinal designs.

Here we report the development of, and initial studies using, the Cognitive Assessment for Neuropsychological Impairments in Dogs (CANID). CANID is a streamlined battery based on the measures developed in Hargrave et al. (2025), with modifications to enable completion in a single study visit. We model relationships between CANID scores and age, assess repeatability at 6- and 12-month intervals, and probe whether repeated testing is associated with practice effects.

Our objective in developing CANID was to facilitate more rapid and streamlined data collection, enabling acquisition of all measures within a single 40–60 minute session while maintaining sensitivity to age-related dysfunction. We therefore began with the three tasks most strongly associated with age in Hargrave et al. (2025), measuring response flexibility, short-term spatial memory, and inhibitory control. Additionally, we refined two of the three tasks to reduce administration time. We also aimed to assess the feasibility of using CANID in longitudinal studies.

Assessment of test-retest reliability is a frequent part of the development of cognitive and psychological measures in humans (Calamia et al., 2013) and has been used in questionnaire measures developed to assess dog behavior, personality, or temperament via owner-report (Duffy & Serpell, 2008; Ley et al., 2009; Salonen et al., 2021; Tiira & Lohi, 2014) and in behavioral tasks designed to quantify dog temperament (Harvey et al., 2016; Turcsán et al., 2018). However, it has historically been rare for animal cognition researchers to do the same when designing cognitive tasks for nonhuman species (Kubinyi & Iotchev, 2020; Schubiger et al., 2020; Shaw & Schmelz, 2017). Few canine cognition studies have quantified the stability of cognitive traits over time, and methods by which repeatability is quantified vary. Some have quantified developmental stability from early life to adulthood (Bray et al., 2021; Junttila et al., 2025) while others focus on consistency within adults (Bognár et al., 2024; Piotti et al., 2022). Repeatability is often under-addressed in studies of animal cognition generally, despite being a crucial element for establishing validity and reproducibility. Cauchoix et al. (2018) conducted a meta-analysis of contextual and temporal repeatability of cognitive measures across many animal taxa, including humans. They demonstrated consistent individual differences in cognition, with a significant yet low average estimate for temporal repeatability. Data from human and nonhuman animal studies compiled by Cauchoix et al. (2018) suggest that cognitive measures may be less repeatable than measures of temperament and personality. Additionally, their meta-analysis suggested negligible learning effects with repeated task exposure and no effect of the delay interval between repeated testing on repeatability.

In one of the few assessments of test-retest reliability in dog cognition, Bognár et al. (2024) reported good repeatability for composite scores and factors related to individual problem solving and learning, but poor repeatability for measures of point-following and sustained visual attention. These results suggest differences in repeatability depend on task type or cognitive domain. The authors also note decreasing repeatability with increasing length of time between tests, especially intervals greater than 2.5 years, which could be attributable to cognitive aging in their study population.

In addition to assessing test-retest reliability, it is important to quantify any improvement in scores due to learning, especially in tests used for longitudinal research (Di Bari et al., 2002). Practice effects (systematic improvement with repeated exposure to a test) can interfere with longitudinal studies of cognition. For example, in human studies, practice effects can obscure detection of mild cognitive impairment (Elman et al., 2018). Knowing the extent to which participants improve with repeated task exposure can allow researchers to account for that systematic improvement in analyses. Conversely, variation in learning across time may reflect aspects of cognitive decline. In tasks where practice effects are normal and expected, the *absence* of improvement with repeated testing (or a lesser degree of improvement) may signal current or future cognitive impairment (Jutten et al., 2020). In animal cognition, learning effects are uncommonly assessed, but it seems that some task types, such as measures focusing purportedly on inhibitory control, may be especially susceptible to improvement with repeat exposures (Loyant et al., 2025; Salomons et al., 2026).

We present data from an ongoing longitudinal study in which 239 dogs underwent baseline CANID testing, with follow-up assessments at six and twelve months (6-month N = 140, 12-month N = 118). Using these data, we estimate 1) relationships between cognitive performance and dog age, 2) repeatability across different time scales, and 3) practice effects associated with repeated testing.

## METHODS

Methodological details regarding study procedures are reprinted with modifications from, GeroScience, 47, Hargrave et al. (2025), “Characterizing dog cognitive aging using spontaneous problem-solving measures: development of a battery of tests from the Dog Aging Project”, with permission from Springer. We provide a comprehensive methods manual and other materials for implementation of these methods in an OSF repository (see “Availability of CANID Methods Manual and Resources”).

### Participants and data collection sites

Data collection occurred at three sites including a university research laboratory (Arizona Canine Cognition Center, Tucson, AZ, USA), a dog daycare facility (Sit! Stay! Play!, Tucson, AZ, USA), and a service dog training center (Canine Companions, Santa Rosa, CA, USA). Demographic information for participating dogs is provided in Table 1. Dogs tested at the Arizona Canine Cognition Center were recruited from a database of local dog owners who had expressed interest in participation in cognitive and behavioral studies. Dogs tested at the dog daycare facility were recruited through informational flyers provided to clients. Dogs tested at the service dog training center were recruited through word of mouth and electronic invitations to dog owners near the data collection site.

### General methods

Dogs participated in a battery of three tasks administered in a single session lasting approximately 40–60 minutes. The three tasks were designed to measure aspects of memory and executive function and included a response flexibility task (Spatial Reversal), a short-term spatial memory task (Delayed Search), and an inhibitory control task (Cylinder). The battery also included a brief assessment of vision and hearing (Sensory Screen). These tasks were identical to or adapted with minor modifications (described below) to reduce time requirements, from Hargrave et al (2025).

A subset of dogs (ESM Table 1) participated in a preliminary version of this battery described in Hargrave et al. (2025) which included the same three tasks plus two additional tasks. For the two tasks that were truncated from the preliminary to the current version, data for these dogs were restricted to the same number of trials to allow direct comparison to data from the current, shorter versions. Detailed methods for each task, presented in order of administration, are described in the subsections below.

All tasks involved a handler who positioned the dog at a start location and timed the trials, and an experimenter who administered the test and recorded data. All sessions were video recorded and behavioral responses were coded in real time. A random subset of ≥ 20% of trials from all tasks were later coded from video by a second observer to assess inter-rater reliability (see “Statistical Analysis”).

All tasks were designed as problem-solving activities in which dogs participated for food rewards. Dogs were not systematically fasted prior to participation. However, owners were informed that it may be helpful to avoid feeding them within a few hours prior to testing and/or to give them a partial rather than a full meal ahead of testing, particularly if their dog was likely to be satiated by 40 small treats. Food rewards were typically small pieces of commercial meat-based dog treats (Jerky Treats, distributed by Big Heart Pet Brand, Orrville, OH) or kibble from the dog’s typical diet, but occasionally varied according to dog preferences or dietary restrictions. In our initial work, an odor control task confirmed that dogs were unable to choose accurately in these tasks based on olfactory cues (Hargrave et al., 2025).

A total of 20 experimenters administered CANID. Some experimenters were trained prior to the beginning of the study, while others were trained on an ongoing basis as needed to support data collection. New experimenters underwent a detailed training process which required study of written protocols, review of videos from prior experiments, observation of trained experimenters, practice sessions implementing protocols, and supervision by a trained experimenter during initial formal data collection. A full methods manual and materials to support training of research staff in CANID implementation is provided on OSF (see “Availability of CANID Methods Manual and Resources”).

Across all tasks, if a dog did not move from the starting position when the experimenter said “okay” (the cue to release the dog), the experimenter repeated the “okay” release cue at 2-s intervals, up to a total of three times. If the dog had not initiated a response (e.g., moved forward to search) by the third release cue, the handler gently nudged the dog from behind to encourage them to move forward from the start line without directing them towards any particular choice location (Delayed Search) or side (Cylinder and Spatial Reversal). If at any point a dog became fearful or anxious during testing and was not easily soothed, we discontinued data collection for that task (Spatial Reversal N = 6, Delayed Search N = 2; no dogs aborted the Cylinder task due to fear). If a dog exhibited fear, anxiety, or stress at the beginning of a session and did not become comfortable within approximately 10–15 min of acclimation, no data collection occurred (N = 6). Likewise, no data collection occurred if a dog was hesitant to consume the food rewards offered freely during acclimation (N = 3).

At the Arizona Canine Cognition Center, owners were present (positioned in a neutral location behind the dog during testing) in addition to the experimenter and handler, but owners were typically absent during testing at the other locations.

**Figure 1:**
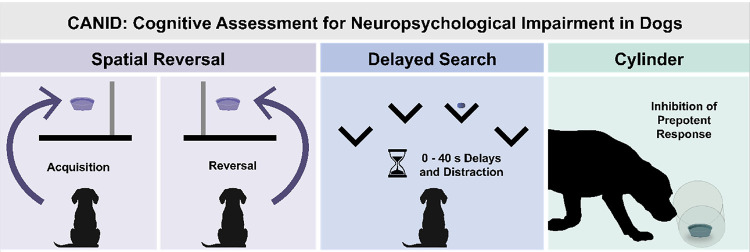
Schematic of the three cognitive tasks in CANID.

The procedures described below are presented in order of administration. Each task consisted of a flexible, unscored warm-ups phase that ensured dogs were comfortable approaching the apparatus and, in the case of the Delayed Search and Cylinder tasks, allowed dogs to learn how to access food from behind v-shaped blinds and within the Cylinder, respectively. The warm-ups phase was followed by a series of scored test trials. Schematics illustrating the three cognitive tasks are shown in Fig. 1.

### Spatial Reversal task

This task measured a dog’s response flexibility, or ability to adapt their behavior to changing circumstances, in the context of a spatial memory task.

#### *Warm-up trial*.

The warm-up trial served to ensure dogs were comfortable approaching and eating near the apparatus. Dogs were positioned at a start line 1.83 m in front of a freestanding 0.91 m × 0.91 m panel. In the warm-up trial, the experimenter showed the dog a treat, placed it into a metal bowl (height = 75 cm, diameter = 180 cm), and then placed the bowl on the ground in front of the panel such that it was visible to the dog. The dog was then released and allowed 20 s to obtain the food reward from the bowl. If dogs were not immediately comfortable approaching on the first attempt of the warm-up trial, they were encouraged to retrieve the treat, and the warm-up trial was repeated until they readily approached and consumed the food without encouragement.

#### *Side choice trial*.

The side choice trial was used to determine the dog’s initial preferred direction of approach. In the side choice trial, the experimenter stood centered behind the panel and held a treat above the panel such that it was visible to the dog. The experimenter then visibly placed the treat into the bowl and lowered the bowl behind the panel, placing it on the ground. The experimenter turned around to face away from the dog as the handler released the dog to search for the food behind the panel, which could be obtained by detouring to either the left or right side of the panel. If a dog did not obtain the reward within 20 s, the trial was repeated, and if the dog failed to obtain the reward within three trials, data collection was discontinued. The side choice trial did not contribute to scoring, but the side from which the dog approached on this trial determined the setup for the baseline phase of the task.

In all subsequent baseline and reversal test trials, the procedure was identical to that of the side choice trial with the exception that a barrier (0.61 m wide × 0.91 m tall) was positioned behind the front panel (Fig. S5), which prevented the dog from accessing the food from one side. The barrier blocking access to the food from one side was not visible to the dog from the dog’s start line.

#### *Baseline test trials*.

For the baseline block, the closed side was the side not initially chosen (i.e., if a dog navigated to the left side of the panel in the side choice trial, the barrier prevented approaches to the right in subsequent trials). The barrier was constructed of wire mesh such that if the dog approached the closed side, they could see (but not access) the food reward, as well as the open path on the opposite side of the apparatus. During baseline test trials, dogs were allowed up to 20 s from the start of the trial to use the open path to access the reward. The trial ended when the dog accessed the reward within the allotted 20 s. During both the baseline phase and the subsequent reversal phase, if a dog approached the closed side but was unable to detour to the open side on their own, the dog was led to the open side by the handler and allowed to obtain the reward. The handler showing the dog how to access food served to maintain motivation to search and equalize dogs’ experience of accessing food at the open side, ensuring that on every trial, if they chose a side, dogs gained experience obtaining the reward via the open path. If the dog did not approach either side of the apparatus (scored as “no choice,” which was neither correct nor incorrect), the dog was not led to the open side, but instead was given a “motivator trial” allowing them easy access to the food placed in a bowl in front of the apparatus (identical to the warm-up trial), followed by a repeat of the test trial on which the no choice occurred. On each trial, we recorded the side first approached, operationally defined as the side on which the dog first placed any paw into a marked proximity zone (43 cm × 20 cm). Before moving on to the next phase, dogs were required to meet a criterion of first approaching the open path in three of four consecutive trials (not including their initial side choice that determined the open side in baseline trials). If a dog did not meet baseline criterion within 20 trials, the Spatial Reversal task was stopped, and the dog continued to subsequent tasks.

#### *Reversal test trials*.

In each reversal block, the barrier was shifted to the opposite end of the panel, closing the path that had been open on the preceding trials, and opening the path on the opposite side. Thus, dogs were required to inhibit the previously successful response and instead employ a new response to access the reward. Trials were repeated in the reversed configuration until the dog chose to first approach the open path in three of four consecutive trials. Upon meeting this criterion, the position of the barrier was reversed again, and another block of trials was administered. This general procedure continued until the dog met the criterion on a total of three reversal blocks, or a total of 20 trials had elapsed during this stage of the task (reversal trials), whichever occurred first. Throughout all phases of the Spatial Reversal task, no delay was imposed between trials; rather, the subsequent trial began as soon as the dog was repositioned at the start line.

#### *Dependent measures*:

1. The total number of blocks the dog completed (the number of blocks in which criterion was met, including the baseline block and up to 3 reversal blocks – range 0–4, lower numbers indicative of worse cognitive functioning)
2. The number of trials in the dog’s single longest block (range 4–20, higher numbers indicative of worse cognitive functioning)
3. The number of unused trials remaining out of 20 possible trials at the conclusion of the task (e.g., 0 for any dog that did not complete three reversals within 20 trials in the reversal phase, lower numbers indicative of worse cognitive functioning)

Dogs who did not meet baseline criterion within the 20 allotted trials and thus did not proceed to the reversal phase (see “Baseline test trials”) were assigned the worst possible score, with a longest block length of 20 trials, 0 trials remaining, and a total of 0 (of 4 possible) phases completed.

We used Principal Component Analysis to generate a Spatial Reversal summary score using these three dependent measures as the input (see Statistical Analysis).

### Delayed Search

This task assessed a dog’s ability to remember the location of a hidden reward across increasing retention intervals, and in the context of distractions.

#### *Warm-up trials*.

The Delayed Search task requires dogs to search for food rewards hidden behind v-shaped blinds placed on the ground. Before beginning test trials, we conducted a warm-up procedure to acclimate dogs to the blinds and the process of searching for food behind them. Across the warm-up trials and the subsequent test trials, treats were positioned at the apex of the baited blind(s) such that they were not visible to the dog unless the dog looked down into the blind from above. During the warm-up, a single blind was positioned in a central location and the experimenter placed a treat behind the blind as the dog watched. The dog was then immediately released to search for the food and was led to the treat by the experimenter if they did not quickly find it on their own. This process was repeated until the dog reliably searched for and located a treat placed behind a single blind. The experimenter then presented the dog with two blinds, positioned equidistantly in front of the dog in a central area of the room, and placed a single treat behind one of the blinds as the dog watched. Again, the dog was immediately released to search for the hidden food, and the experimenter helped the dog to locate the reward if they did not quickly find it independently. This process was repeated until the dog independently approached the baited blind first on at least two occasions. After dogs learned to search for rewards behind blinds, we briefly familiarized them with the procedure that would be used as a distraction in test trials (see below). The experimenter walked rapidly towards the dog and placed a treat on the ground directly in front of the dog, then immediately turned around and returned to their starting position.

#### *Test trials*.

Four blinds were arranged in an array 2.74 m from the dog’s starting point, such that all blinds were equidistant from the dog. On each trial, the experimenter showed the dog a treat, and as the dog watched, placed it behind one of the blinds. Dogs were released immediately once the experimenter returned to their starting position after baiting in the first four test trials (no delay). In the remaining eight test trials, dogs were required to wait for a specified duration before being released to search. We used three fixed delays of 10 s (4 trials), 20 s (2 trials), and 40 s (2 trials), which were administered sequentially. The first two test trials were identical to the no-delay trials except that dogs were required to wait 10 s before searching. In the remaining six test trials, we implemented a distraction to prevent dogs from using non-mnemonic strategies such as sustained attention or body orientation towards the hidden reward (Krichbaum et al., 2021). In these distraction trials, during the delay (after placing a treat behind a blind), the experimenter walked towards the dog and delivered a small food reward on the ground in front of the dog, then returned to their starting position. To consume this reward, dogs were required to lower their heads, breaking any possible fixation on the blinds. Additionally, the dogs’ view of the blinds became momentarily obscured by the experimenter’s body. For each test trial, dogs were given 20 s to search for the hidden reward. We recorded the first location that the dog searched, operationally defined as the dog’s snout entering the corner of the blind or the dog’s head being positioned above the area behind the blind with a downward head orientation. If the first location the dog searched was incorrect, we allowed the dog to continue searching until 20 s had elapsed, or until the reward was located. If the dog searched in at least one location but did not locate the treat within 20 s, to prevent declines in motivation associated with failure to obtain a reward, the handler led them to the baited location and allowed them to eat the treat. However, if the dog did not search in any blinds (scored as “no choice”), the dog was not shown the food but was instead returned to the start line and participated in a “motivator trial” in which the experimenter placed a single blind directly in front of the dog, baited it, and released the dog immediately. If the “no choice” occurred on a distraction trial, the motivator trial incorporated the delivery of a distraction treat as well but still had only one blind available for the dog to choose from. Following the motivator trial, the trial in which the “no choice” occurred was repeated. If the dog made four “no choices” during the test trials, the task was discontinued (N = 3 dogs across timepoints, of which all three provided sufficient data for imputation of remaining trials).

The reward location was balanced across trials (three times in each location) and was never the same on consecutive trials. The reward location followed the same predetermined order for all dogs.

#### *Dependent measures*.

For this task, we assessed the proportion of correct trials within each trial type (i.e., no delay, 10-s delay, 10-s delay with distraction, 20-s delay with distraction, 40-s delay with distraction). For a small subset of dogs who did not make choices on all trials (N = 15), we imputed missing data using predictive mean matching, implemented in the **m**ultiple **i**mputation through **c**hained **e**quations (mice) R package (Buuren & Groothuis-Oudshoorn, 2011).

We used Principal Component Analysis to generate a Delayed Search summary score using proportion correct on each trial type as the input (see Statistical Analysis).

### Cylinder

This task measured a dog’s motor inhibition and response flexibility in the context of changing task demands.

#### *Warm-up trials*.

Warm-up trials used an opaque black cylinder (26.5 cm in length) with an opening at one end (diameter 22.5 cm) that was positioned 1.24 m from the dog. The experimenter acclimated the dog to approaching the cylinder by performing one trial in which they placed a small silicone bowl (height = 2.5 cm, diameter = 11.5 cm), in front of the cylinder and allowed the dog to eat from it. One “leading trial” was then conducted in which the experimenter guided the dog to the open end of the cylinder by allowing them to follow a food lure into the opening of the cylinder. If a dog was cautious when approaching the bowl/apparatus in either of these warm-up trials or hesitant to put their snout into the opening of the cylinder during the leading trial, the dog was encouraged to get the treat and the trial was repeated until the dog would readily follow the food lure into the cylinder.

#### *Opaque cylinder trials*.

Subsequently, dogs participated in five familiarization trials in which they watched the experimenter place a food reward inside the cylinder, then were released to access the food from the open end of the cylinder independently (without a lure). The open side of the cylinder was always on the dog’s left (experimenter’s right). This phase allowed dogs to repeatedly practice the behavior of accessing the treat inside the cylinder and helped ensure that the strategy to access the treat from the open side was fluent before the inhibitory control test trials (see below).

To prevent declines in motivation associated with failure to obtain a reward, if the dog touched the cylinder but did not obtain the food within 20 s, the experimenter guided them to the correct side and allowed them to eat the food from within the cylinder. If 20 s elapsed before the dog either successfully retrieved the food or contacted the apparatus, the trial was marked as “no response.” Dogs were not guided to the treat following a “no response” trial, but instead participated in a “motivator trial,” which for this stage of the task (opaque trials) was identical to the leading trial from warm-ups in which dogs follow a food lure into the opening. After the “motivator trial,” the test trial on which the “no response” occurred was repeated. All scored responses reflected the dog’s independent actions without the experimenter’s assistance.

#### *Inhibitory control test trials*.

These test trials were identical to opaque cylinder trials with the exception that the food reward was placed inside a transparent cylinder, allowing the subject to see the food reward through the front of the apparatus. A successful response (detouring to the open end of the cylinder) therefore required subjects to resist the prepotent response of approaching the visible food directly. Four test trials were performed. We recorded whether dogs touched the exterior of the cylinder with their snout or paw prior to accessing the food inside the cylinder. As before, if a dog touched the cylinder but did not access the food within 20 s, the experimenter guided them to the opening and allowed them to eat the food. If dogs did not access the reward or contact the apparatus within 20 s (“no response”, not scored), they were not guided to the food, but instead participated in a “motivator trial” (the experimenter bowl placed in front of the cylinder as in the first warm-up trial), followed by a repeat of the “no response” trial. If “no response” trials occurred four total times across the opaque trials and inhibitory control test trials, data collection for this task was discontinued (N = 4 dogs across all timepoints).

#### *Dependent measure*.

We used the proportion of trials in which dogs obtained the reward without first contacting the exterior of the cylinder in inhibitory control trials. Dogs who made responses on three or fewer inhibitory control trials were excluded from analysis (N = 3 dogs across all timepoints).

### Sensory screen

Since older dogs may experience age-related hearing or vision loss, we conducted a brief sensory screening to assess responsiveness to visual and auditory stimuli. Failure on the vision screening was treated as an exclusion criterion given that all tasks required dogs to process visual stimuli. Failure on the auditory screening was not used as an exclusion criterion given that processing of auditory stimuli was not essential for task participation and these dogs would still attend to the experimenter’s movements and readily search for food when released. In the vision screening, the experimenter stood 1 m in front of the dog and dropped a cotton ball (25 cm diameter) from the dog’s eye level to the floor in front of the dog. The experimenter then kneeled 1 m in front of the dog and 0.67 m to the side, and using a finger, flicked the cotton ball such that it passed in front of the dog from the dog’s left to right, and then repeated this procedure from the other side, flicking the cotton ball from the dog’s right to left visual field. The dog passed the visual screening if they followed the motion of the cotton ball with their head and/or eyes. All dogs passed the vision screening. The auditory screening consisted of an experimenter pressing a training clicker on the opposite side of their body from the dog such that the clicker was obscured from the dog’s view and approximately 0.33 m from the dog’s head. The dog passed the auditory screening if they showed an immediate (within ~ 1 s) observable response to the clicker on either side, including but not limited to a rapid change in head orientation or an ear flick on the side of the stimulus. N = 11 dogs did not pass the auditory screening during one or more testing timepoints.

### Development of CANID from preliminary study

The battery presented by Hargrave et al. (2025) included five tasks that were administered across two study visits, each approximately 30–60 minutes. Our goal in developing CANID was to create an even shorter instrument that still robustly captured age-related cognitive dysfunction in dogs. To do so, we retained the three tasks with the strongest associations with age in Hargrave et al. (2025) and made minor modifications to some tasks to enable more efficient administration, without compromising sensitivity to age-related variation.

In the Spatial Reversal task, we reduced the total number of trials from 30 to 20 based on retrospective exploratory analyses of data from Hargrave et al. (2025), which indicated that highly similar summary scores could be obtained using a reduced number of trials. Additionally, by using a lower trial cap we aimed to reduce potential for fatigue in older dogs who, relative to younger animals, typically require more trials to complete this task. Second, based on observations that some geriatric dogs failed to complete the baseline within 20 trials, a possible indication of profound cognitive impairment, we modified the scoring for this task to include data from baseline trials as an additional block in the summary score (previous scoring considered only subsequent reversal blocks, not the baseline block, when calculating the total number of blocks completed and the number of trials in the longest block).

The Cylinder task was modified to eliminate the reversal phase described in Hargrave et al. (2025). This change reduced the overall length of the Cylinder task and was informed by exploratory analyses which revealed stronger age associations on the inhibitory control trials preceding the reversal component of the task.

The Delayed Search task was retained from Hargrave et al. (2025) without modification.

Because the modifications described above only involved a shortening (reduction of total trial numbers, or elimination of later task stages) of procedures described by Hargrave et al. (2025), it was possible to rescore data collected under the original procedures using the updated CANID scoring protocol.

For a summary of task changes from Hargrave et al. (2025), see ESM Table 2.

### Availability of CANID Methods Manual and Resources

To facilitate and encourage use of CANID by other researchers, we developed a detailed guide to implementing these protocols, including a methods manual, video library, data collection tools, and code for scoring and analysis. These materials are available in an OSF repository (https://osf.io/n7jt9/overview).

### Repeat Testing

Baseline CANID scores were obtained at dogs’ initial study visits. To assess retest reliability and potential practice effects, a subset of dogs underwent repeat testing at six and twelve months following baseline at the same test location as their baseline visit (with few exceptions – 4 dogs originally tested at Sit! Stay! Play! experienced at least one follow-up timepoint at the Arizona Canine Cognition Center). Ninety-one dogs have data for all three study timepoints (see ESM Table 3 for details).

### Statistical Analysis

Analyses were performed using R Statistical Software (R Core Team, 2025). To assess inter-rater reliability, a second rater coded a randomly selected ≥ 20% of trials. Inter-rater reliability (Cohen’s Kappa) was excellent for all variables used in scoring (ESM Table 4).

Our statistical models made use of two types of summary scores, generated using the procedures from Hargrave et al. 2025 and described below. Using all baseline data, we first generated task-level summary scores for each subject, reflecting their overall performance on a specific task. We then generated overall CANID summary scores, which provide an aggregate measure of performance across the different tasks. The Spatial Reversal and Delayed Search task scores were derived from principal components analyses with the dependent measures for each task as the input data and scores on the first component used as the task summary score. The Cylinder score did not require this approach as it was a single measure (proportion correct). To generate overall (cross-task) summary scores we used a similar approach incorporating task-level summary scores as the input data, and component scores from the first component as an overall CANID score. We standardized task and overall scores before performing statistical analyses, including applying a Yeo-Johnson transformation to Spatial Reversal and Delayed Search task scores to normalize their distributions.

To generate task scores and overall CANID scores for data from follow-up timepoints, we used the predict() function from the psych package (William Revelle, 2025) to project data from six- and twelve-month retests into the same principal component space defined using baseline data.

For dogs missing a single task summary score (due to aborting the entire task or having insufficient task data: n = 10 dogs at baseline, 1 at 6-month retest, and 3 at 1-year retest), we imputed their score on the missing task when generating overall CANID scores, but did not include imputed data when analyzing task-specific repeatability and practice effects. Imputation used the linear regression-based method ‘norm.predict’ from the mice package (Buuren & Groothuis-Oudshoorn, 2011) with the observed task scores and test location as predictors. For dogs with missing data on more than one task (6 dogs at baseline), we did not generate an overall CANID score.

To assess the effect of repeated test administration and the association between age and cognitive score, we fit linear mixed-effects models using the lme4 package (Bates et al., 2015) with fixed effects for dog age at test (modelled using a second-degree orthogonal polynomial to accommodate non-linear age effects), follow-up timepoint (e.g., baseline, 6-month, 1-year), sex, and test location, and a random effect for dog ID. Models were fit for both the overall CANID score and separately for each of the three individual tasks (Spatial Reversal, Delayed Search, Cylinder).

As estimates of repeatability, we report bivariate intraclass correlation coefficients between timepoints as well as adjusted ICC values from mixed models incorporating effects for age and other covariates and ICC from an unadjusted (intercept-only) model, comparable to R (repeatability) which has been reported in other animal cognition and behavior studies (Table 3).

## RESULTS

Scores on all three tasks loaded positively on a single component (Spatial Reversal: 0.81, Delayed Search: 0.64, Cylinder: 0.69) that explained 51.2% of variation. We used scores on this component as the overall CANID score. Details regarding raw measures for the three component tasks are presented in supplemental material (ESM Tables 5 and 6).

**Figure 2:**
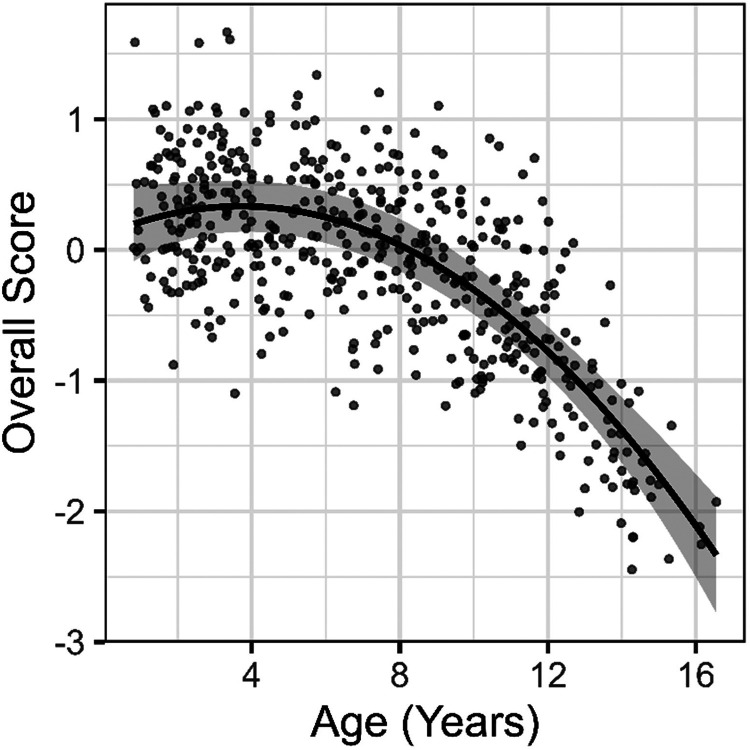
Relationship between age and overall CANID scores estimated from a linear mixed-effects model. Points reflect partial residuals from the mixed model (adjusted for all model terms except age). The gray shaded region represents the 95% confidence interval

**Figure 3:**
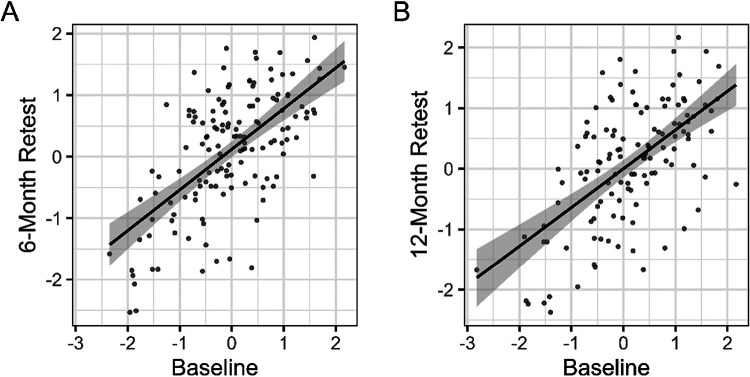
Repeatability of overall CANID scores at A) 6 months compared to baseline and B) 1 year compared to baseline. Shaded regions indicate 95% confidence intervals

Age accounted for 27% of the variance in overall scores (partial *R*^*2*^ = 0.27), corresponding to a large effect (Cohen’s *f*^*2*^ = 0.43; see Fig. 2 and Table 2). A likelihood ratio test comparing models with and without age (modeled as a second-degree polynomial), indicated that age significantly improved model fit (χ2(2) = 114.79, p < .001). The model-based adjusted ICC for overall CANID scores was 0.45, indicating moderate repeatability when accounting for age. Pairwise ICCs between timepoints and ICC from an unadjusted (intercept-only) model were higher (Fig. 3, Table 3), indicating that some within-individual consistency is attributable to strong, systematic age effects rather than individual differences that are independent of age. Modest improvements in overall scores were evident at the six-month (*b* = 0.23, 95% CI [0.10, 0.37]) but not twelve-month retest (*b* = 0.04, 95% CI [−0.10, 0.19]).

There were significant effects of both the first- and second-order age terms for all individual tasks (ESM Fig. 1, ESM Tables 7–9). The effect of timepoint on score varied by task. We observed improvement in Spatial Reversal scores at the six-month retest (*b* = 0.20, 95% CI [0.03, 0.37]), but not the twelve-month retest (*b* = 0.02, 95% CI [−0.16, 0.21]). Dogs’ scores on the Delayed Search task were similar to baseline at the six-month retest (*b* = 0.01, 95% CI [−0.14, 0.16]) but lower at the twelve-month retest (*b* = −0.24, 95% CI [−0.41, −0.08]). On the other hand, a slight but enduring practice effect was observed for the Cylinder task (*b* = 0.28, 95% CI [0.13, 0.44]). Repeatability as assessed by ICCs from an intercept-only model (i.e., without accounting for age effects) for all tasks was high relative to previously reported repeatability in animal cognition (Cauchoix et al., 2018) (Table 3, ESM Fig. 2).

## DISCUSSION

In this study, we introduced the Cognitive Assessment for Neuropsychological Impairments in Dogs (CANID) and assessed its suitability for longitudinal aging research by measuring test-retest reliability, practice effects, and sensitivity to age-related cognitive dysfunction. We found that overall CANID scores, as well as scores on all three of the underlying subtests, have robust negative associations with age and that these scores are repeatable across 6- and 12-month periods, with a modest, short-term practice effect at 6 months that was not observed at 12 months. Measures used in task scoring have excellent interrater reliability (ESM Table 4) indicating strong to near-perfect agreement (McHugh, 2012). The combination of sensitivity to cognitive dysfunction, temporal repeatability, high interrater reliability, minimal learning effects with repeated task exposure, and potential for efficient administration makes CANID a robust and promising tool for longitudinal studies of cognition in dogs.

An important element of the current study was measurement of test-retest reliability at 6- and 12-month intervals. Because there are many ways in which researchers estimate repeatability of cognitive and behavioral measures, direct comparisons across studies can be challenging. However, the repeatability of overall CANID scores exceeds meta-analytic estimates of behavioral/personality consistency in nonhuman animals—dogs (mean r = 0.43, Fratkin et al., 2013), temporal repeatability of cognitive task performance across taxa (mean adjusted R = 0.28, Cauchoix et al., 2018)—and approximates retest reliability in human neuropsychological measures (r ~ 0.7, Calamia et al., 2013) even when controlling for variation explained by age, which non-aging-focused studies seldom address. Furthermore, while some studies report that repeatability differs by task type (Ashton et al., 2022), all three CANID tasks exhibited substantial repeatability (Table 3).

Practice effects are normal and expected in many cognitive tests. We observed that repeated exposure at six months from baseline resulted in small overall performance gains that appear to attenuate by twelve months, such that the twelve-month retest score is not statistically different from baseline. At the six-month retest, dogs’ overall scores improved on average + 0.23 standard deviations from baseline, a magnitude comparable to the average retest gains of ~ 0.24 standard deviations found in a large meta-analysis of human cognitive tests (Calamia et al., 2012). Our observed pattern of retest performance, short-term improvement which then dissipates, could be due to a practice effect that is then offset by aging-related decline at twelve months. In human studies, older individuals show smaller practice effects than younger individuals (Calamia et al., 2012) and Alzheimer’s patients may lack practice effects altogether or exhibit less improvement with repeated exposure than healthy controls (Duff et al., 2024). Worse performance in Delayed Search at twelve months (ESM Table 8) may allude to some decline being measurable over this time span, but further work modeling trajectories separately in young and old dogs is needed. In the Cylinder task, dogs’ raw scores exhibited a slight but enduring performance boost (ESM Table 9), with six- and twelve-month retest scores averaging 9 percentage points higher than baseline. It is possible that this stable increase in task performance after baseline reflects increased experience with retrieving a reward from a transparent barrier after their first exposure to this task. Indeed, as animals in transparent detour-reaching tasks gain additional experience that these barriers are solid and impenetrable, their performance can improve (van Horik et al., 2018). In sum, CANID scores demonstrate small and mostly transient practice effects which are within the range typically observed in human neuropsychological testing.

Finally, we found a significant effect of test location on performance after controlling for important covariates such as age. Specifically, dogs tested at Canine Companions performed better than those tested at an on-campus research center or at a dog daycare (Table 2). These differences appear to be driven by significantly better performance on tasks requiring inhibition: Cylinder (motor inhibition of prepotent response) and Spatial Reversal (suppression of previously learned spatial response) (ESM Table 7 and Table 9). In contrast, while no overall score differences were observed for dogs tested at Sit! Stay! Play!, these dogs performed slightly, but significantly, worse on the Cylinder task compared to dogs tested at the Arizona Canine Cognition Center (ESM Table 9), potentially reflecting the influence of a higher arousal testing environment which can negatively affect inhibitory control. In this sample, dogs tested at Canine Companions predominantly represent dogs who 1) began service dog training but who were released from the program, 2) underwent some service dog training and became part of the organization’s breeding program, or 3) were retired or actively working service dogs at the time of testing. Thus, the better performance observed in this group could relate to temperament differences, such as lower baseline arousal, which is known to influence executive function in dogs (Bray et al., 2015), training history (Bray et al., 2023), which was likely more extensive for these dogs compared to others in our sample, or the relative genetic homogeneity of a group of dogs bred for behavioral and health traits suitable for assistance dog work. While some studies show benefits of training and cognitive stimulation for cognitive functioning in older dogs (Bray et al., 2023; Chapagain et al., 2017; Milgram et al., 2006), others find no effect (Chapagain et al., 2020). The concept of cognitive reserve and its protective effects with respect to cognitive aging are well-documented in humans (Nelson et al., 2021; Opdebeeck et al., 2016; Pettigrew & Soldan, 2019). Future research should further examine whether factors such as training history and lifetime cognitive enrichment (both potentially analogous to ways cognitive reserve is quantified in humans) could prevent or slow cognitive aging in dogs.

An important next step will be to assess CANID’s ability to detect within-individual change across aging, particularly in senior dogs. We observed that scores within 12 months of baseline testing were largely stable in a mixed-age population. Following dogs over longer periods and modeling longitudinal changes in relation to age will be critical for assessing CANID’s suitability for measuring cognitive decline. Additionally, if CANID is to be used in clinical studies where individual outcomes are of interest, it will be important to determine whether an individual is declining beyond what can be expected by measurement error, so important benchmarks such as minimum detectable change should be established.

Future research should also explore additional aspects of validity, such as whether CANID scores agree with other measures of cognitive dysfunction. Doing so can be a way of assessing both construct validity, specifically convergent validity (i.e., agreement between CANID and other, typically questionnaire-based scales) and ecological validity (i.e., owner-reported measures of everyday cognitive function). Because laboratory studies suggest that Canine Cognitive Dysfunction (CCD) is preceded by a stage of mild cognitive impairment (Studzinski et al., 2006), it is possible that CANID scores could be predictive of dogs’ future scores on established CCD scales rather than convergent with concurrent CCD status. Longer term studies accompanied by cognitive dysfunction questionnaires and/or veterinary diagnostics are needed to address this question. CANID’s sensitivity to mild dysfunction in mid-life, earlier than the typical ages at which signs of CCD appear (Madari et al., 2015; Neilson et al., 2001; Salvin et al., 2010), supports the possibility that CANID may detect preclinical decline. Future work may also combine CANID with CCD assessments to help uncover if CANID can differentiate normal cognitive aging from pathological cognitive decline. In CANID, decline is apparent beginning around the age of 8 years, whereas in questionnaire-based assessments of CCD, the probability of exceeding the diagnostic threshold does not increase sharply until approximately 12 years (Bray et al., 2023). If CANID is indeed capable of detecting earlier and subtler signs of impairment, it could serve as a more powerful tool for evaluating the effects of lifestyle, environmental, and genetic factors on cognitive aging, and for assessing responses to pharmacological, dietary, or behavioral interventions designed to prolong cognitive health.

In developing CANID, our goal was not only to create a sensitive and efficient tool for assessing cognitive aging in companion dogs, but also to make this tool broadly accessible to the research community. To facilitate replication and adoption, we have made all materials publicly available, including a detailed methods manual, apparatus specifications, training videos, standardized data sheets, and R code for scoring (https://osf.io/n7jt9/overview). By lowering logistical and methodological barriers, we hope CANID can be readily implemented across laboratories and in diverse studies focused on cognitive aging in dogs. Widespread use of a shared, open, and standardized functional assessment will enable more direct comparisons across studies, accelerate discovery, and ultimately strengthen our capacity to understand and intervene in cognitive aging.

## Figures and Tables

**Table 1.** Selected demographic information for dogs participating in the development and testing of CANID.

	Baseline			6-month Retest			12-month Retest		
	N	Mean	SD	N	Mean	SD	N	Mean	SD
Age (years)	239	7	4	140	7.6	4	118	7.3	3.9
Weight (kg)	239	22	11	140	22	11	118	23	11
Sex	239			140			118		
Spayed female	109	46%		61	44%		53	45%	
Neutered male	109	46%		69	49%		57	48%	
Intact female	12	5%		3	2%		1	1%	
Intact male	9	4%		7	5%		7	6%	
Test Location	239			140			118		
Arizona Canine Cognition Center	116	49%		67	48%		46	39%	
Canine Companions	31	13%		1	1%		21	18%	
Sit! Stay! Play!	92	33%		72	51%		51	43%	
Breed Category	239			140			118		
Mixed breed	121	51%		59	42%		54	46%	
Purebred	110	49%		81	58%		64	54%	

**Table 2.** Results of a linear mixed-effects model estimating the association between age and overall CANID score.

Predictor	Beta	95% CI	p-value
Timepoint			
Baseline	—	—	
6-Month Retest	0,23	0.10,0.37	<0.001
12-Month Retest	0.04	−0.10, 0.19	0.5
Test Age (Years)			
1st degree	−11	−13, −8.9	<0.001
2nd degree	−5.2	−7.1, −3–3	<0.001
Sex			
Female	—	—	
Male	0.07	−0.11, 0.25	0.4
Test Location			
Arizona Canine Cognition Center	—	—	
Canine Companions	0.65	0.36, 0,94	<0.001
Sit! Stay! Play!	−0.07	−0–26, 0.12	0.5

**Table 3:** Repeatability metrics and pairwise ICCs for overall CANID score and individual CANID tasks. 95% confidence intervals are provided in square brackets

Overall Score	Adjusted ICC [95% CI]	Intercept-Only ICC	Baseline/6-Month ICC	Baseline/12-Month ICC	6-Month/12-Month ICC
	0.45 [0.29, 0.58]	0.63 [0.49, 0.74]	0.63 [0.51, 0.72]	0.59 [0.47, 0.70]	0.57 [0.42, 0.69]
Spatial Reversal	0.29 [0.12, 0.47]	0.43 [0.26, 0.58]	0.41 [0.26, 0.54]	0.52 [0.37, 0.63]	0.38 [0.19, 0.54]
Delayed Search	0.45 [0.29, 0.61]	0.51 [0.34, 0.64]	0.51 [0.38, 0.62]	0.47 [0.32, 0.59]	0.57 [0.42, 0.69]
Cylinder	0.46 [0.28, 0.59]	0.53 [0.34, 0.66]	0.59 [0.48, 0.69]	0.36 [0.19, 0.50]	0.48 [0.31, 0.62]

## Data Availability

Data used in this manuscript, and accompanying code for analysis, can be found in the project’s OSF repository (https://osf.io/n7jt9/overview).

## Dog Aging Project Consortium authors

Joshua M. Akey^14^, Rozalyn M. Anderson^15,16^, Elhanan Borenstein^17,18,19^, Marta G. Castelhano^20,21^, Amanda E. Coleman^22^, Kate E. Creevy^23^, Matthew D. Dunbar^3^, Virginia R. Fajt^24^, Jessica M. Hoffman^25^, Erica C Jonlin^26,27^, Matt Kaeberlein^26,28^, Elinor K. Karlsson^29,30,^ Kathleen F. Kerr^31^, Jing Ma^32^, Stephanie McGrath^33,34^, Natasha J Olby^35^, May J Reed^36^, Audrey Ruple^37^, Stephen M. Schwartz^38,39^, Sandi Shrager^40^, Noah Snyder-Mackler^41−43^, M. Katherine Tolbert^23^

^14^ Lewis-Sigler Institute for Integrative Genomics, Princeton University, Princeton, NJ, USA

^15^ University of Wisconsin Madison, Madison, WI

^16^ GRECC William S Middleton Memorial Veterans Hospital, Madison WI

^17^ Department of Clinical Microbiology and Immunology, Sackler Faculty of Medicine, Tel Aviv University, Tel Aviv, Israel

^18^ Blavatnik School of Computer Science, Tel Aviv University, Tel Aviv, Israel

^19^ Santa Fe Institute, Santa Fe, NM, USA

^20^ Cornell Veterinary Biobank, College of Veterinary Medicine, Cornell University, Ithaca, NY, USA

^21^ Department of Clinical Sciences, College of Veterinary Medicine, Cornell University, Ithaca, NY, USA

^22^ Department of Small Animal Medicine and Surgery, College of Veterinary Medicine, University of Georgia, Athens, GA, USA

^23^ Department of Small Animal Clinical Sciences, Texas A&M University School of Veterinary Medicine & Biomedical Sciences, College Station, TX, USA

^24^ Department of Veterinary Physiology and Pharmacology, Texas A&M University College of Veterinary Medicine & Biomedical Sciences, College Station, TX, USA

^25^ Department of Biological Sciences, Augusta University, Augusta, GA, USA

^32^ Department of Laboratory Medicine and Pathology, University of Washington School of Medicine, Seattle, WA, USA

^27^ Institute for Stem Cell and Regenerative Medicine, University of Washington, Seattle, WA, USA

^28^ Department of Oral Health Sciences, University of Washington, Seattle, WA, USA

^29^ Bioinformatics and Integrative Biology, University of Massachusetts Chan Medical School, Worcester, MA, USA

^30^ Broad Institute of MIT and Harvard, Cambridge, MA, USA

^31^ Department of Biostatistics, University of Washington, Seattle, WA, USA

^32^ Division of Public Health Sciences, Fred Hutchinson Cancer Research Center, Seattle, WA, USA

^33^ Department of Clinical Sciences, College of Veterinary Medicine and Biomedical Sciences, Colorado State University, Ft. Collins, CO

^34^ Brain Research Center at Colorado State University

^35^ Department of Clinical Sciences, College of Veterinary Medicine, North Carolina State University, Raleigh, NC, USA

^36^ Department of Medicine, Division of Gerontology and Geriatric Medicine, University of Washington School of Medicine, Seattle, WA

^37^ Department of Population Health Sciences, Virginia-Maryland College of Veterinary Medicine, Virginia Tech, Blacksburg, VA, USA

^38^ Epidemiology Program, Fred Hutchinson Cancer Research Center, Seattle, WA, USA

^39^ Department of Epidemiology, University of Washington, Seattle, WA, USA

^40^ Collaborative Health Studies Coordinating Center, Department of Biostatistics, University of Washington, Seattle, WA, USA

^41^ School of Life Sciences, Arizona State University, Tempe, AZ, USA

^42^ Center for Evolution and Medicine, Arizona State University, Tempe, AZ, USA

^43^ School for Human Evolution and Social Change, Arizona State University, Tempe, AZ, USA
